# Patient’s attitudes and perceptions around attending oncology consultations following surgery for colorectal cancer: A qualitative study

**DOI:** 10.12688/f1000research.134816.1

**Published:** 2023-06-19

**Authors:** Yoshan Moodley, Shona Bhadree, Laura Stopforth, Shakeel Kader, Steven Wexner, Jacqueline van Wyk, Alfred Neugut, Ravi Kiran

**Affiliations:** 1 University of KwaZulu-Natal, Durban, KwaZulu-Natal, South Africa; 2 Stellenbosch University, Stellenbosch, Western Cape, South Africa; 3 Cleveland Clinic Florida, Weston, Florida, USA; 4 University of Cape Town, Rondebosch, Western Cape, South Africa; 5 Columbia University, New York, New York, USA

**Keywords:** Attitudes, Perceptions, Post-procedural, Consult, Surgery, Colorectal cancer.

## Abstract

**Background:** The oncology consultation following surgery for colorectal cancer (CRC) is usually the first step in the receipt of chemotherapy. Non-compliance with this consultation results in non-receipt of recommended chemotherapy, when appropriate, and worse clinical outcomes. This study sought to explore South African patients’ attitudes and perceptions around attending scheduled oncology consultations following their CRC surgery.

**Methods:** Semi-structured qualitative interviews were conducted with patients who had surgery for CRC at a quaternary South African hospital and who had to decide whether they would return for an oncology consultation. The “Model of health services use” informed the design of the interview guide, which included questions on factors that impact health seeking behavior. Demographics of participants, CRC disease stage, and compliance with scheduled oncology consultations were also collected. Descriptive statistics were used to analyse the quantitative data, while deductive thematic analysis was used to analyse the qualitative data.

**Results:** Seven participants were interviewed. The median age was 60.0 years and four participants (57.1%) were female. Black African, White, and Asian participants accounted for 85.7% of the study sample. Most participants had stage III CRC (71.4%). The oncology consultation no-show rate was 14.3%. Participant’s knowledge and beliefs around CRC proved to be an important predisposing factor that influenced follow-up decisions. Family support and religion were cited as important enabling factors. Travel costs to the hospital and frustrations related to the clinic appointment booking/scheduling process were cited as important disabling factors. Lastly, the participant’s self-perceived need for additional oncology care also appeared to influence their decision to return for ongoing oncology consultation after the initial surgery.

**Conclusion:** Several contextual factors can potentially influence a patient’s compliance with a scheduled oncology consultation following CRC surgery. A multipronged approach which addresses these factors is required to improve compliance with oncology consultations.

## Introduction

Colorectal cancer (CRC) incidence has steadily increased in South Africa, and it is now one of the most significant causes of cancer-related morbidity and mortality in the country. ^1^ The initial recommended treatment for CRC is surgery; however, adjuvant chemotherapy might also be necessary in the context of advanced CRC. ^2^ Evidence from clinical trials demonstrates the benefits of receiving adjuvant chemotherapy, notably improved disease-free survival and improved overall survival rates. ^3^ The initiation of chemotherapy is preceded by an oncology consultation, at which a patient is assessed as a possible candidate for treatment by an oncologist. ^4^ A treatment plan might also be communicated by the oncologist to patients who are deemed good candidates for adjuvant chemotherapy. ^4^ Therefore, the oncology consultation is seen as a crucial step to the possible receipt of chemotherapy. The published literature reports that 15-34% of eligible CRC patients will not participate in a scheduled oncology consultation, ^5^^,^^6^ and, therefore, will likely not end up receiving their recommended chemotherapy. Given the growing public health problem posed by CRC in South Africa and the implications if patients do not access and discuss treatment options with an oncologist, this study sought to explore South African patients’ attitudes and perceptions around attending scheduled oncology consultations following surgery for CRC.

## Methods

### Study design and setting

**Figure 1.**
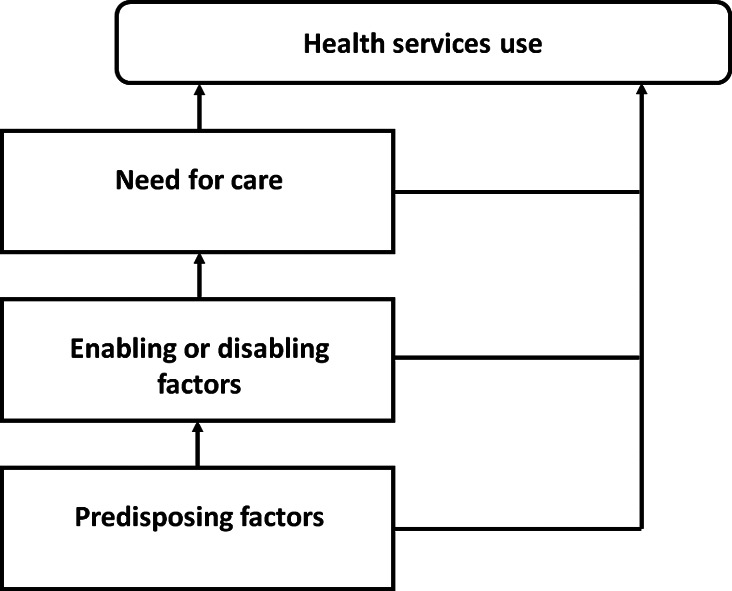
Outline of the “Model of health services use”.

This was an exploratory, qualitative study. The theoretical framework adopted for this study was the “Model of health services use”. ^7^ As outlined in Figure 1, this model proposes that an individual’s use of healthcare services is based on the interaction of factors which predispose him/her to use healthcare services, factors which facilitate or hinder the use of healthcare services, and factors that drive the need for care. This study was conducted at the Inkosi Albert Luthuli Central Hospital (IALCH) in Durban, South Africa. The hospital is a government-funded facility which offers specialist healthcare services, including oncology care, to the population of the KwaZulu-Natal Province on the eastern seaboard of South Africa. IALCH is the only quaternary-level hospital in the province, and patients predominantly access health care services at the facility after being referred from lower-level healthcare facilities.

### Study population

The study population included adult (aged >18 years old) CRC patients with stage III/IV disease who had undergone surgery at IALCH during the period 13 April 2022-12 June 2022, and who needed to decide on whether they would return for their scheduled oncology clinic consultation. A convenience sampling strategy was used to identify and invite eligible patients to participate in this study. Informed consent was obtained from all study participants. Participants were enrolled in this study (hereafter referred to as “the study sample”) until data saturation was reached and no new information was collected for the qualitative analysis.

### Data collection

Information on each participant’s age, gender, and race was collected directly from their medical records and entered on a Microsoft Excel spreadsheet. A trained research assistant conducted semi-structured qualitative interviews at the bedside, just before the participants were discharged from hospital after their surgery. The interview questions, shown in Table 1, explored the three main components of the “Model of health services use” – predisposing factors, enabling or disabling factors, and factors which drive the need to access healthcare services. ^7^ Interviews were conducted in English or the local isiZulu Language, where appropriate. Predisposing factors can include patient demographics and health beliefs. Given the qualitative nature of this research, a decision was made to restrict the interview questioning around “predisposing factors” to the participant’s health beliefs. Broadly, enabling factors include patient socioeconomic factors (high level of education, being employed), social factors (psychosocial support mechanisms in place), and health system-related factors (access to healthcare information and efficient delivery of healthcare). Disabling factors encompass the converse of enabling factors. The need for care can be considered as perceived (patient) or evaluated (healthcare provider). Since the recommended treatment for stage III/IV CRC usually involves a combination of surgery followed by adjuvant chemotherapy, ^8^ it was assumed that all healthcare providers would consider there to be a need amongst CRC patients for an oncology consultation following their cancer surgery. As such, the interview questioning around factors which drive the need to access healthcare was restricted to the participant’s perspective (perceived need for care) rather than the healthcare provider’s perspective (evaluated need for care). All interviews were recorded electronically and took about 30 minutes to complete. The content from the interviews was transcribed shortly after the interview was completed and entered onto a Microsoft Excel spreadsheet for coding and the subsequent data analysis. In addition, the medical records of participants were screened to establish compliance with instructions to return for an oncology consultation at IALCH.

**Table 1.** Key questions explored during the semi-structured interview.

“Model of health services use” component	Interview questions
Predisposing factors	• Tell me about your beliefs about this cancer?
Enabling or disabling factors	• What factors will make it difficult or easier for you to come back to the oncology clinic for your consultation with the oncologist? • Are any of the factors more important to you and your family? • How will each factor influence your decision to continue with your treatment? • In what way can the doctor/nurse or other professional(s) at the hospital help to ensure that you come for your next treatment opportunity?
Need for care	• Do you think that you will come back for oncology care?

### Data analysis

Descriptive statistics were used to summarize the key characteristics of the study sample. The results of the descriptive statistical analysis are presented as medians (interquartile range) or as frequencies (%). Deductive thematic analysis was used to analyse the data from the semi-structured qualitative interviews. ^9^ The deductive thematic analysis followed six steps ^10^: familiarisation with the data (listening to the recorded interviews), coding of the data (generating broad labels for important features of the data), generating themes (collating the coded data under similar themes), reviewing themes (assessing the relevance of individual themes and whether some themes might need to be collapse together or split into two or more themes), defining themes (conducting and writing a detailed analysis of each theme), and writing up the findings from the qualitative analysis (integrating the analytic narrative and the data extracts from the interviews). Both the descriptive statistics and the deductive thematic analysis were performed using Microsoft Excel.

## Results

### Descriptive statistical analysis

A total of seven participants were interviewed. An overall description of the study sample is provided in Table 2. The median age of the study sample was 60.0 years, and there were four female participants (57.1%). There were similar numbers of Black African, White, and Asian participants (two participants from each of these demographic groups, accounting for 85.7% of the study sample). Two participants had stage IV CRC (28.6%), while the remaining five participants had stage III CRC (71.4%). There was one participant who was a no-show for the oncology consultation (14.3%).

**Table 2.** Overall demographic characteristics of study participants (N=7).

Characteristic	Summary statistic
***Age in years***	
Median (interquartile range)	60.0 (50.5-64.0)
***Gender***	
Male, n (% of N)	3 (42.9)
Female, n (% of N)	4 (57.1)
***Race***	
Black African, n (% of N)	2 (28.6)
Asian, n (% of N)	2 (28.6)
White, n (% of N)	2 (28.6)
Mixed, n (% of N)	1 (14.3)
***Cancer disease stage***	
Stage III	5 (71.4)
Stage IV	2 (28.6)
***Returned for oncology consultation***	
Yes, n (% of N)	6 (85.7)
No, n (% of N)	1 (14.3)

### Qualitative analysis

Information on the characteristics of the individual participants that comprised the study sample is provided in Table 3. The findings of the deductive thematic analysis are reported as three core themes based on the “Model of health services use” - predisposing factors, enabling or disabling factors, and factors driving the need for care. ^7^

**Table 3.** Demographic characteristics of individual study participants (N=7).

Participant	Age in years	Gender	Race	Cancer disease stage	Returned for oncology consultation
P1	48	Female	Asian	IV	Yes
P2	53	Female	Black African	III	Yes
P3	66	Male	Asian	III	Yes
P4	60	Female	Black African	III	Yes
P5	72	Male	White	III	Yes
P6	62	Male	Mixed	III	No
P7	48	Female	White	IV	Yes

### Predisposing factors

Some participants possessed knowledge that informed their understanding and beliefs around CRC, as indicated below. Some knew that cure was possible and were able to identify more complex risk factors for CRC, such as genetics and pre-cancerous colonic polyps. The need for timely treatment of pre-cancerous colonic polyps was also acknowledged by one patient.

P1: “I know it is curable. I really don’t have specific beliefs but since I always had constipation all my life, so maybe it has something to do with constipation. Well, I won’t say it is related to my family genes since I don’t know my family history.” [48; Female; Asian]

P4: “Yes, there are beliefs that I have, what I know is they say if you have small polyps, they might predispose you into developing colon cancer if they are not removed.” [60; Female; Black African]

On the other hand, when asked about what their beliefs were regarding CRC, there were also some participants who did not have a clear understanding (or no understanding at all in some instances) of how CRC develops, the disease process, and its associated risk factors.

P5: “I don’t know, I really have no idea. However, they say it can pass from one person to another - my mother had skin cancer, my grandmother had either breast cancer or bone marrow cancer, either than that my father had no cancer, my brothers and sisters have no cancer and none of our children have cancer. Um, I don’t know.” [72; Male; White]

P2: “I would be lying if I say I have any belief that is associated with cancer, but I normally hear people saying you can develop cancer from smoking, and I don’t know how true it is.” [53; Female; Black African]

P6: “No, I have no idea.” [62; Male; Mixed race]

### Enabling and disabling factors

Family support and religion were cited by several participants as important enabling factors. Family members were a source of strength and psychosocial support for some study participants.

P4: “Family support, I think it’s best for my family to know what I am going through so that I can get all the support I need from them.” [60; Female; Black African]

P5: “Regarding my family, it is all the love I am receiving from them that keeps me going.” [72; Male; White]

P6: “The family support influence will be very strong for me in such a way that they will make sure I come for my appointments.” [62; Male; Mixed race]

P7: “My daughter works in Durban, so she makes sure she drops and pick me up at the hospital, so there is not much of an inconvenience. She pays for my medical bills.” [48; Female; White]

Participants were motivated by prayer, religion, and words of encouragement from their local religious leaders.

P3: “Religion is very important; it is important in everyday life. Yesterday I could not pray following surgery as I was still in pain.” [66; Male; Asian]

P5: “It’s my God that opened the doors for me to be here. If he did not want me to be here, he would have closed the doors.” [72; Male; White]

P7: “Well, my pastor in church believes that I should be spokesperson for people with cancer, of which I would like to do when but once I am healed or fully recovered. Maybe I can be a volunteer in at a hospital well, I will see. I would like to give back.” [48; Female; White]

All but one of the study participants did not attend their scheduled oncology consultation. Travel costs to the hospital for the oncology consultation were cited as an important disabling factor by one of the participants. She stated that she was the only breadwinner in her family, and that she had to balance the need to seek treatment for her CRC against possible unpaid leave from her employment.

P2: “The cost of getting here. The main issue is the money for transport since I only work for certain days, and the most difficult part is that I only earn R2000 which is not enough to support my family. If I can have enough money that will make my situation easy. Money is the main issue. Money is the most important factor since I am the only one who is working, everyone is relying on me. Since I am here in hospital, I must make sure that I ask for the letter of attendance otherwise I won’t even get paid. I work as a cleaner, so my money is not enough, as I end up borrowing money to make it to the hospital for my appointments.” [53; Female; Black African]

Another participant, who resided a great distance away from IALCH also cited transportation costs as a disabling factor. He also stated that besides the costs associated with his transport to IALCH, he would have to seek financial assistance from his family to secure accommodation in Durban while he waited for his return trip home.

P3: “The cost of getting to Durban from Newcastle. My family supports me financially to get accommodation in Durban.” [66; Male; Asian]

Some participants felt that healthcare providers could do more to facilitate their compliance with scheduled oncology consultations. These frustrations were mostly related to the clinic appointment booking/scheduling process.

P1: “They must make sure that we are registered (at the hospital admissions desk) for our consultation. It happens every time when I get to the clinic by 5 am, to find out that I am not registered and had to go back to register and to the clinic and wait again.” [48; Female; Asian]

P2: “Maybe if they can book my appointments towards month end, when I have money for transport, that will help me not to have stress of borrowing money from people.” [53; Female; Black African]

P3: “I am not sure, maybe they can send me reminder few days before my appointment.” [66; Male; Asian]

There were certain participants who merely required encouragement to come back for a scheduled oncology consultation, such as in the case below.

P6: “Well, they can encourage me. For example - if you come, we will do this for you and we going to clear this.” [62; Male; Mixed race]

Other participants requested honest conversations with their healthcare providers regarding their care and reasons for why the oncology appointments were being scheduled.

P4: “They must tell you why it is important to come for the follow-up.” [60; Female; Black African]

P5: “They must be straight forward; in other words, the healthcare professionals must let you know if there are greater or lesser chances of surviving.” [72; Male; White]

### Factors driving the need for care

The most important factor driving the need for care was the self-perceived need for additional oncology care following surgery. Two participants appeared to perceive themselves as still being at risk for CRC following surgery (and hence requiring the recommended additional oncology treatment).

P2: “I will make sure I come for ongoing care, because at the end of the day I need help.” [53; Female; Black African]

P7: “I will come back, even the doctors told me that I will definitely be coming back here for ongoing care.” [48; Female; White]

There was one participant who, due to misconceptions around the aggressiveness of this cancer, perceived that he was no longer at further risk following surgery, and did not require additional treatment.

P3: “I don’t think so, because once they have removed the cancer, I should be fine. This cancer is not aggressive.” [66; Male; Asian]

## Discussion

Although it is based on a small sample size, the observed oncology consultation no-show rate in this South African study was comparable with the range of no-show rates reported in the published literature from high-income countries, ^5^^,^^6^ suggesting that non-compliance with scheduled oncology consultations amongst CRC patients is indeed a global problem. However, this finding takes on added significance in the South African context for two reasons: Firstly, CRC is an emerging public health concern in South Africa, even amongst younger populations, ^1^^,^^11^ and the expectation is that there would also be a growing trend in the number of individuals who require surgery and additional oncologic treatment for CRC. Secondly, the South African public healthcare sector is resource-constrained when compared with public health systems in high-income countries, ^12^^,^^13^ and missed oncology consultations would have implications for efficient utilisation of clinical oncology services, as well the downstream consequences of incomplete CRC treatment on patient clinical outcomes.

The published literature reports that a large portion of cancer patients do not receive oncology care due to their refusal of treatment, ^14^ highlighting the importance of patient-centered research in this population. The current study improves our understanding of context-specific factors that influence a patient’s decision to default on a scheduled oncology consultation. The study has identified several key contextual factors of interest, all of which fall within the scope of the established “Model of health services use” and are amenable to modification. These factors must be considered by South African public health specialists when tailoring a response to this challenge.

A clear understanding of CRC and its treatment is required to enable patients to make informed decisions regarding their subsequent oncology care. ^15^^,^^16^ Public health campaigns targeting CRC are generally lacking in African countries. ^17^ Therefore, South African CRC patients have limited access to vetted educational materials on the subject. Most patients in the current setting will likely receive information about their disease via a direct conversation with the healthcare workers who are also treating them, or their information will be based on discussions with their friends and family. In the current study, several participants expressed misconceptions around the causes of CRC and how the disease progresses. Not only does this finding emphasize the shortcomings in how healthcare workers communicate information on CRC to afflicted patients, but it also questions the quality of information delivered through other traditional sources. There is much scope to improve patient information to the general public through the well-designed educational materials on CRC. As per the characteristics of the study sample, the CRC population of KwaZulu-Natal Province is racially and culturally diverse. ^11^ Accordingly, any new educational materials or awareness campaigns for CRC will need to be culturally relevant and accessible in isiZulu, the most common dialect in the region. Knowledge and awareness campaigns have proven to be effective at increasing the use of CRC disease screening, ^18^ and this approach will likely have a similar impact on other aspects of the cancer treatment pathway, including oncology consultation attendance.

The perceived need for healthcare can be an important factor which prompts patients to seek healthcare services. ^19^^,^^20^ While it is encouraging that most study participants had still perceived themselves as being at risk for CRC following surgery (and there still being a need for additional oncology care), patient educational materials on CRC would also be of benefit in raising awareness around the consequences of incomplete oncology treatment in those who do not feel the need for further oncology care due to misconceptions related to the seriousness of the disease.

Communication of CRC information between healthcare workers and patients, particularly information regarding the importance of oncology care, can be improved through educational interventions for healthcare workers. In-person workshops to improve physician’s communication practices with cancer patients have been well-received. ^21^^,^^22^ Formal training in communication practices would also ensure that relevant information on CRC treatments is communicated to patients and delivered in a standardised manner.

Up to 80% of cancer patients desire a qualitative prognosis (i.e. “Will I die from this disease”) from their attending physician. ^23^ This conversation can be difficult for the physician for several reasons: news of a poor prognosis can be depressing for a patient and could lower their motivation for additional care; the misconception that involvement of Hospice care will reduce patient survival; fear that the prognosis may be incorrect, and the possible medicolegal implications thereof; whether it is culturally appropriate to deliver news of a poor prognosis to some patients; and the stress that delivering this news places on the physician himself/herself. ^24^ Approaches to assist physicians having this complex and difficult discussion with their patients have been reported. ^25^ The first step in this process involves establishing whether the patient wants to know more on their prognosis, and how much they would like to know. For patients who do request more information on their prognosis, the content of the discussion is then negotiated; information is provided; the patient’s (and family’s) response to the news is acknowledged; and the patient’s understanding of the discussion should be assessed. ^25^

The important role played by family in providing motivational support to a cancer patient during their treatment journey has long been acknowledged. A recent systematic review reported that being married (versus being unmarried) was associated with a significantly lower odds of treatment refusal amongst CRC patients. ^26^ In addition to motivational support, participants in the current study also stated that family members were crucial in providing financial support or directly facilitating their attendance to hospital visits. These important roles played by family members should be encouraged and strengthened. However, there might be instances where a patient does not have any family members to support them during their cancer treatment journey. In such instances, close friends or peers may assume this supportive role. Peers are cancer survivors who have been cured of cancer, and are thus well-placed to provide motivation, advice, and information to other cancer patients who are just beginning their treatment journeys. ^27^

Spirituality is considered a fundamental component of holistic person-centered care. ^28^ The published literature demonstrates the benefits of religion and spirituality in the context of cancer, empowering patients to accept their illness and allowing them to deal with it in a way that is both positive and purposeful. ^29^^,^^30^ This notion was indeed confirmed in the current study, with many participants citing religion as a source of strength and a coping mechanism as they battle their disease. It is therefore crucial that the spiritual needs of CRC patients be assessed and addressed throughout the cancer treatment journey. There are existing tools that have been used to assess the spiritual needs of cancer patients, ^30^ however these originate in populations which are culturally and racially different from the South African population. The existing tools may, however, be less accurate in assessing the spiritual needs of CRC patients in the South African setting, thereby necessitating the development of a locally relevant tool for this purpose. Oncology units should also look to work closely with community-based religious groups, to ensure that patients who do require spiritual support are able to draw on support from community-based resources.

Even in settings where efficient transportation networks exist, distance to a healthcare facility is known to impact compliance with cancer treatment. ^31^ It is therefore unsurprising that transportation costs were cited by participants in this study as a barrier to attending an oncology consultation. This barrier could potentially be addressed in one of two ways: Firstly, transportation to the hospital on the day of the oncology consultation could be arranged through provision of a dedicated hospital bus service to patients. ^32^ Secondly, it might be possible to decentralise the oncology consultation to a lower-level healthcare facility which is closer to a patient’s place of residence, ^33^ thereby facilitating attendance of the consultation. Both proposed solutions are likely to have financial implications for the resource-constrained South African public healthcare system. However, this would be worthwhile in the long term as patients with CRC would be linked to care more efficiently, thereby averting some of the undesired clinical outcomes associated with this disease. ^34^

Forgetting or being completely unaware of a scheduled clinic appointment are common reasons for missed clinic appointments and are not unique to oncology care. ^35^ Many participants in this study expressed the need to be reminded about their oncology consultation. Ownership of mobile phones amongst the South African population is high when compared with other African countries, ^36^ and the opportunity exists to leverage this technology to reduce the possibility of missed oncology appointments in CRC patients. Pre-appointment short message service (SMS) alerts have been used with much success (reduced the “did not attend” response by nearly 50%) in the United Kingdom at a breast cancer clinic. ^37^ However, there are several factors which must be considered before a similar intervention for CRC patients can be implemented in South Africa. These factors include illiteracy, patient confidentiality, and loss or theft of mobile phones. ^38^ Allowing for flexibility in the patient appointment scheduling system might be appreciated by patients who are employed and who may not be able to take time off from work on their initially scheduled appointment date. ^39^ Lastly, identifying gaps in the patient admission system and addressing these gaps would help alleviate the frustrations around patient workflow between the hospital admissions department and the oncology clinic. ^40^

This research was not without limitations. The sample size was small, and the quantitative findings on oncology consultation no-shows should be interpreted with caution. Although the study participants were representative of the CRC population in KwaZulu-Natal Province, ^11^ they are likely to differ from CRC patients in other South African provinces and the findings of this research might therefore not be entirely applicable to CRC patients across all the South African provinces. This study did not include CRC patients from the South African private healthcare sector, who might also have different attitudes and perceptions around attending oncology consultations following their surgery. In addition, this study enrolled participants over a short time period and it is also possible that there might be seasonal differences in oncology consult no-show rates or patient responses as to why they would possibly not attend an oncology consultation.

In conclusion, this study has identified several contextual factors based on the “Model of health services use” which can potentially influence a patient’s compliance with a scheduled oncology consultation following CRC surgery. Bearing in mind that these contextual factors are unlikely to occur in isolation, a multipronged approach which addresses these factors is required to improve CRC patient’s compliance with oncology consultations. Despite the useful information yielded by this study, the findings should be confirmed with additional research which attempts to address some of the limitations that have been declared.

## Data Availability

Open Science Framework: Patient’s attitudes and perceptions around attending oncology consultations following surgery for colorectal cancer, https://doi.org/10.17605/OSF.IO/9T5BV. ^41^ This project contains the following underlying data:
• Dataset.xlsx • Interview transcripts: Interview_transcripts.xlsx Open Science Framework: ‘SRQR’ checklist for ‘Patient’s attitudes and perceptions around attending oncology consultations following surgery for colorectal cancer’, https://doi.org/10.17605/OSF.IO/9T5BV. ^41^ Data are available under the terms of the Creative Commons Attribution 4.0 International license (CC-BY 4.0).
