# Supplementary material for: Patient’s attitudes and perceptions around attending oncology consultations following surgery for colorectal cancer: A qualitative study
Source: F1000Res. 2023 Jun 19;12:698. [Version 1] doi: 10.12688/f1000research.134816.1 (PMC10762288; doi:10.12688/f1000research.134816.1)
Supplement: Peer review history

# Reviewer report: Reviewer response for version 1

**DOI:** 10.5256/f1000research.147899.r229118

**Published:** 2024-01-19

**Authors:** Rajesh Balkrishnan

**Affiliations:** 1 University of Virginia School of Medicine, Charlottesville, VA, USA

This is a qualitative study assessing perceptions around getting oncologic consultations following colorectal cancer surgery in a group of South African patients. Overall the study is well conducted and detailed in the report. The paper will be enhanced if the authors can spend more time in introduction and discussion discussing the importance of follow up care after CRC surgery and what type of interventions may be needed to increase post operation follow up rates.

Is the work clearly and accurately presented and does it cite the current literature?

Yes

If applicable, is the statistical analysis and its interpretation appropriate?

Yes

Are all the source data underlying the results available to ensure full reproducibility?

Yes

Is the study design appropriate and is the work technically sound?

Yes

Are the conclusions drawn adequately supported by the results?

Partly

Are sufficient details of methods and analysis provided to allow replication by others?

Yes

Reviewer Expertise:

Cancer epidemiology

I confirm that I have read this submission and believe that I have an appropriate level of expertise to confirm that it is of an acceptable scientific standard.

# Reviewer report: Reviewer response for version 1

**DOI:** 10.5256/f1000research.147899.r229113

**Published:** 2024-01-02

**Authors:** Haniee Chung

**Affiliations:** 1 The Johns Hopkins University School of Medicine, Baltimore, USA

A simple study design to answer a very practical and important question. This study highlights the universal discrepancy that exists between providers' understanding and patients' perception of appropriate cancer care. No amount of evidence based clinical service will make up for cultural/social/economic hurdles that act as barriers to care if a patient is unwilling or unable to overcome them. Studies like this begin to close this gap. 

 A particularly interesting and consideration is the recognizable global phenomenon in the rise in CRC in LMICs where previously CRC rates were low or unknown. This, in conjunction with the rise in young CRC in HICs raise very thought provoking questions regarding not only the pathophysiology underlying CRC development, but also the barriers to care that lead to higher stage at diagnosis. 

 To emphasize the urgency and importance of adjuvant systemic treatment, please consider including language and findings of studies that have established the importance of initiating treatment within 8-12 weeks postoperatively. In clinical practice, patients may no-show, but also present late- after this recommended window of treatment. Adding this to the suggested improvement in patient education may also be useful.

Is the work clearly and accurately presented and does it cite the current literature?

Partly

If applicable, is the statistical analysis and its interpretation appropriate?

Yes

Are all the source data underlying the results available to ensure full reproducibility?

Yes

Is the study design appropriate and is the work technically sound?

Yes

Are the conclusions drawn adequately supported by the results?

Yes

Are sufficient details of methods and analysis provided to allow replication by others?

Yes

Reviewer Expertise:

Colorectal cancer outcomes, Global surgery education

I confirm that I have read this submission and believe that I have an appropriate level of expertise to confirm that it is of an acceptable scientific standard.

## References

[1] : Adjuvant chemotherapy for stage III colon cancer: does timing matter?. *Dis Colon Rectum* .2011;54(9) : 10.1097/DCR.0b013e318223c3d6 1082-9 10.1097/DCR.0b013e318223c3d6 21825887

[2] : Short-term outcomes of Early versus conventional adjuvant chemotherapy in stage III colon cancer: randomized clinical trial. *BJS Open* .2023;7(4) : 10.1093/bjsopen/zrad064 10.1093/bjsopen/zrad064 37439066 PMC10338899

# Reviewer report: Reviewer response for version 1

**DOI:** 10.5256/f1000research.147899.r204249

**Published:** 2023-09-14

**Authors:** Shanaz Ghuman

**Affiliations:** 1 Community Health Studies, Durban University of Technology, Durban, KwaZulu-Natal, South Africa

Thank you for the opportunity to review the manuscript on patients attitudes and perceptions around attending oncology consultations following surgery for colorectal cancer: a qualitative study.  The paper is well written, and despite a small sample size of 7 participants the results align with previously published studies/research. The Health Services Use Model is appropriate, and 3 components were presented namely predisposing factors, enabling or disabling factors for compliance with oncology consultation and need for care. The results are presented and reported in tables for qualitative data; thematic analysis for qualitative data define the participants interview responses. The authors conclude that in order to improve oncology consultations, multifaceted approaches are integral to support patients with CRC.

Is the work clearly and accurately presented and does it cite the current literature?

Yes

If applicable, is the statistical analysis and its interpretation appropriate?

Yes

Are all the source data underlying the results available to ensure full reproducibility?

Yes

Is the study design appropriate and is the work technically sound?

Yes

Are the conclusions drawn adequately supported by the results?

Yes

Are sufficient details of methods and analysis provided to allow replication by others?

Yes

Reviewer Expertise:

epidemiology, risk behaviours, public/community health, medicinal plants, community development, administration/management, microbiology

I confirm that I have read this submission and believe that I have an appropriate level of expertise to confirm that it is of an acceptable scientific standard.
